# A BOLD Perspective on Age-Related Neurometabolic-Flow Coupling and Neural Efficiency Changes in Human Visual Cortex

**DOI:** 10.3389/fpsyg.2013.00244

**Published:** 2013-05-03

**Authors:** Joanna Lynn Hutchison, Ehsan Shokri-Kojori, Hanzhang Lu, Bart Rypma

**Affiliations:** ^1^School of Behavioral and Brain Sciences, University of Texas at DallasRichardson, TX, USA; ^2^Department of Psychiatry, University of Texas Southwestern Medical CenterDallas, TX, USA; ^3^Advanced Imaging Research Center, University of Texas Southwestern Medical CenterDallas, TX, USA

**Keywords:** fMRI, BOLD, CBF, CMRO_2_, hypercapnia, aging, neurometabolic-flow coupling, neural efficiency

## Abstract

Age-related performance declines in visual tasks have been attributed to reductions in processing efficiency. The neural basis of these declines has been explored by comparing the blood-oxygen-level-dependent (BOLD) index of neural activity in older and younger adults during visual task performance. However, neural activity is one of many factors that change with age and lead to BOLD signal differences. We investigated the origin of age-related BOLD changes by comparing blood flow and oxygen metabolic constituents of BOLD signal. Subjects periodically viewed flickering annuli and pressed a button when detecting luminance changes in a central fixation cross. Using magnetic resonance dual-echo arterial spin labeling and CO_2_ ingestion, we observed age-equivalent (i.e., similar in older and younger groups) fractional cerebral blood flow (ΔCBF) in the presence of age-related increases in fractional cerebral metabolic rate of oxygen (ΔCMRO_2_). Reductions in ΔCBF responsiveness to increased ΔCMRO_2_ in elderly led to paradoxical age-related BOLD decreases. Age-related ΔCBF/ΔCMRO_2_ ratio decreases were associated with reaction times, suggesting that age-related slowing resulted from less efficient neural activity. We hypothesized that reduced vascular responsiveness to neural metabolic demand would lead to a reduction in ΔCBF/ΔCMRO_2_. A simulation of BOLD relative to ΔCMRO_2_ for lower and higher neurometabolic-flow coupling ratios (approximating those for old and young, respectively) indicated less BOLD signal change in old than young in relatively lower CMRO_2_ ranges, as well as greater BOLD signal change in young compared to old in relatively higher CMRO_2_ ranges. These results suggest that age-comparative studies relying on BOLD signal might be misinterpreted, as age-related BOLD changes do not merely reflect neural activity changes. Age-related declines in neurometabolic-flow coupling might lead to neural efficiency reductions that can adversely affect visual task performance.

## Introduction

The human visual system exhibits age-related changes that lead to changes in the efficiency of information processing. Whereas structural changes are prominent in the periphery (i.e., the cornea, iris, lens, and vitreous and aqueous humors; e.g., Owsley and Sloane, 1987; Michaels, 1993), second- and third-order brain regions in the visual pathway appear to be less affected by aging. Specifically, structural studies of lateral geniculate nucleus (LGN) suggest age-equivalent (i.e., similar in older and younger groups) neuron density and minimal changes in size, and functional imaging studies suggest minimal age-related changes in LGN-cell response properties (Ahmad and Spear, 1993; Spear et al., 1994). Striate cortex similarly undergoes minimal age-related structural changes in neuron density and size (Haug et al., 1984; Vincent et al., 1989); functional changes in this region generally indicate sparing of LGN and V1 function. For example, mechanisms of chromatic contrast adaptation and color-coding have been determined to be largely unchanged with age (Elliott et al., 2012). Despite the apparent age-equivalence of these visual-pathway structures and at least some psychophysical functions, performance declines are often observed in visual tasks. Older adults’ ability to switch between percepts in ambiguous figures is reduced compared to younger adults (e.g., Aydin et al., 2013), as is their ability to perceive shape from texture (e.g., Weymouth and McKendrick, 2012) and to detect objects in complex displays (e.g., Plude and Doussart-Roosevelt, 1989; Hommel et al., 2004; Bennett et al., 2012; Scialfa et al., 2012).

Age-related performance declines in visual tasks have been attributed to reductions in processing efficiency (e.g., Welford, 1981; Grady et al., 1994; Grady, 1996; Speranza et al., 2001). For instance, in a study investigating age-related pattern detection changes, Speranza et al. (2001) presented stereoscopic stimuli with varying degrees of background noise. They observed performance decrements for older adults when noise was introduced into the visual stimuli. Older adults’ performance was not affected differentially by binocular cues or internal noise, nor was their performance related to filter bandwidth changes. Instead, a decrement in processing efficiency, as measured by the signal energy necessary for a subject to detect a signal in a noisy background, was found to be responsible for age-related elevations in both binoptic and dichoptic processing thresholds.

The neural basis of such age-related performance declines has been explored by comparing the blood-oxygen-level-dependent (BOLD) index of neural activity in older and younger adults during visual task performance (e.g., Ross et al., 1997; Buckner et al., 2000; Huettel et al., 2001; Pasley et al., 2007; Ances et al., 2009; Hutchison et al., 2012). BOLD activity is measured by the hemodynamic response function (HRF, the time-response function that describes the relationship between neural and vascular activity; Gössl et al., 2001) using functional magnetic resonance imaging (fMRI). Age-related alterations in BOLD activity across cortex and tasks are often interpreted as age-related alterations in neural activity (e.g., Grady, 2002; Hedden and Gabrieli, 2004; Rajah and D’Esposito, 2005; Nyberg and Bäckman, 2011). Such a straightforward interpretation is complicated by the fact that neural activity is only one of several factors that can change with age and lead to BOLD signal differences (Moeller et al., 1996; Davis et al., 1998; Hoge et al., 1999a; Rypma and D’Esposito, 2000, 2001; D’Esposito et al., 2003; Iadecola, 2004; Rypma et al., 2005; Leontiev et al., 2007; Restom et al., 2007; Ances et al., 2009; Motes et al., 2010).

Hyder (2004) demonstrated that fractional changes in cerebral metabolic rate of oxygen (ΔCMRO_2_) closely approximate neural activity (measured by extracellular recording) during sensory stimulation. Thus, ΔCMRO_2_ would be preferable to the BOLD response as an index for neural activity. However, ΔCMRO_2_ is more expensive, time-intensive, and difficult to acquire, and thus many researchers must rely on the BOLD response as a proxy for neural activity. The BOLD signal is dependent upon structural brain integrity as well as physiological processes that change with age such as cerebral blood flow (CBF; D’Esposito et al., 2003; Buxton et al., 2004). Age-related changes in neurometabolic-flow coupling and vascular dynamics, triggered by a multiplicity of factors such as disease and developmental changes in astrocytic activity, have been observed that could bias estimations of neural activity with BOLD signal and its relationship to behavior (D’Esposito et al., 1999, 2003). For example, an age-related reduction in resting state CBF has been observed under circumstances of increased CMRO_2_ when accounting for parenchyma volume, yielding a decrease in venous oxygenation (Y_v_) with increased age (Lu et al., 2011). This finding suggests that if the supply of blood flow to cortical regions does not keep pace with age-associated increases in metabolic demand, then venous oxygenation is decreased. Additionally, cerebrovascular reactivity (CVR) decreases with age (Ances et al., 2009; Lu et al., 2011; Gauthier et al., 2012). That is, under conditions of activation, blood vessels respond less to changes in metabolic demand. Decreases in Y_v_ and decreases in CVR yield a relatively hypoxic cellular environment for older adults, which is a condition that has been shown to lead to increases in CBF and CMRO_2_ (Xu et al., 2012; but, see Mintun et al., 2001) and could therefore alter the BOLD response.

The BOLD signal is strongly affected by vascular coupling to neural metabolic activity (see D’Esposito et al., 2003; Iadecola, 2004; Cauli and Hamel, 2010). Neural activity leads to fractional increases in cerebral perfusion of oxygenated blood (ΔCBF) that exceed fractional increases in oxygen metabolic rate (ΔCMRO_2_) for active neurons, leading to increases in the BOLD response (also known as the T2* MR signal; Ogawa and Lee, 1990; Ogawa et al., 1992). However, because ΔCBF and ΔCMRO_2_ are not always coupled across cortex and tasks, the ΔCBF/ΔCMRO_2_ ratio and BOLD signal can be affected by the variability in these constituent components, leading to substantial regional differences (cf. Vafaee and Gjedde, 2000; Lu et al., 2004; Vafaee and Gjedde, 2004; Tuunanen and Kauppinen, 2006; Tuunanen et al., 2006; Chiarelli et al., 2007a,b; Ances et al., 2008; but, see Leenders et al., 1990). Mechanisms posited to mediate these differences include variation in oxygen metabolic requirements across cortex (Tuunanen et al., 2006), oxygen delivery, or blood flow alterations under certain conditions or in certain cortical areas (Vafaee and Gjedde, 2000; Lu et al., 2004), variable neurometabolic-flow coupling (Vafaee and Gjedde, 2004), and the accurate estimation of *M* (an index of maximal BOLD responding; Chiarelli et al., 2007a). Regardless of the precise mechanism behind this variability, the end result is that the BOLD response can be difficult to interpret across healthy, young cortex, and it can be even more complicated to interpret when making comparisons between younger and older groups (cf. D’Esposito et al., 2003).

We hypothesized that the relationship between activation-induced ΔCBF and ΔCMRO_2_ in primary visual cortex is fundamentally altered by the process of aging such that the BOLD response might differentially index neural activity in older compared to younger individuals. We further hypothesized that age-related changes in visual task performance – that is, in reaction times (RTs) – are related to changes in the ΔCBF/ΔCMRO_2_ ratio. Few studies have extended concepts of neurometabolic-flow coupling differences to hypotheses of age-related variation in BOLD activity and behavior (Hutchison et al., 2012; Mohtasib et al., 2012). Our study focuses on these basic relationships within distinct sensory cortical areas, minimizing the complexities of functional activity related to higher cognition. To assess the extent to which differences can occur independently in ΔCBF and ΔCMRO_2_ across the cortex, we additionally assessed these relationships within motor cortex. Finally, we performed a simulation to assess effects of age-related neurometabolic-flow coupling changes on the BOLD response.

The use of dual-echo Arterial Spin Labeling (ASL; i.e., calibrated fMRI) technology allows for the separation of vascular and neural factors and permits the resolution of age-related BOLD signal ambiguities because the ASL signal (echo 1) is dependent on CBF while the BOLD signal (echo 2) is dependent on both CBF and CMRO_2_ (cf. Davis et al., 1998; Hyder et al., 2000). In studies using calibrated fMRI, some results have suggested that the ASL and BOLD signals do not have the same signal-to-noise ratio (Wong et al., 2000), potentially affecting the calculation of ΔCMRO_2_. Collection of hypercapnic (Davis et al., 1998) or hyperoxic (Chiarelli et al., 2007b) data in tandem with calibrated fMRI ameliorates this concern to the extent that it allows for scaling of the BOLD response to account for differences in the signal-to-noise ratio. Previous work in both animals (e.g., Kida et al., 2000) and humans (e.g., Davis et al., 1998) has shown the components of calibrated fMRI to be complementary to one another in elucidating the relationship between vasculature, neural activation, and the BOLD response (see Brown et al., 2007; Hoge, 2012).

In one study (Hutchison et al., 2012), we sought to observe age-related neurometabolic-flow coupling changes in visual cortex using calibrated fMRI and hypercapnia (cf. Hoge et al., 1999a,b; Pasley et al., 2007; Ances et al., 2009). The present paper extends this work by describing our investigation of neurometabolic-flow coupling changes within both visual and motor cortex. Additionally, we used simulation techniques to determine effects of reduced vascular responsivity on ΔCBF/ΔCMRO_2_ and BOLD. Subjects periodically viewed flickering annuli and pressed a button as quickly as possible upon noticing changes in the luminance of a central fixation cross (cf. Pasley et al., 2007). Functional signal changes in visual cortex were assessed using both fixation and parafoveal stimulation as baseline conditions of interest. Functional signal changes in motor cortex were assessed in conjunction with button presses. Hypercapnic administration allowed for the calculation of *M* and thus allowed for the calculation of ΔCMRO_2_ and ΔCBF/ΔCMRO_2_ (Leontiev and Buxton, 2007; Leontiev et al., 2007) within both visual and motor cortices.

Within visual cortex, we observed age-equivalent ΔCBF in the presence of age-related increases in ΔCMRO_2_. Reductions in ΔCBF responsiveness to increased ΔCMRO_2_ in the elderly led to paradoxical age-related decreases in BOLD – that is, increases in ΔCMRO_2_, a close index of neural activity, would be expected to result in increases in the BOLD response, but we found the opposite. Age-related ΔCBF/ΔCMRO_2_ ratio decreases were related to RT, suggesting that age-related slowing observed in visual processing tasks results from less efficient neural cell assemblies. Within motor cortex, we found a different pattern of evidence. That is, we found age-related increases in both ΔCBF and ΔCMRO_2_ for older participants, but ΔCBF/ΔCMRO_2_ ratios were not significantly different between younger and older groups. The relatively impoverished responsivity of ΔCBF for older adults still led to an age-related decrease in BOLD. We speculated that such reduced vascular responsiveness to neural metabolic demand would lead to variation in ΔCBF/ΔCMRO_2_ with variation in task demand. A simulation of BOLD relative to ΔCMRO_2_ for lower and higher neurometabolic-flow coupling ratios (approximating those for old and young, respectively) indicated less BOLD signal change in old than young in relatively lower CMRO_2_ ranges (i.e., reduced levels of CMRO_2_ relative to baseline), as well as greater BOLD signal change in young compared to old in relatively higher CMRO_2_ ranges (i.e., increased levels of CMRO_2_ relative to baseline). This simulation illustrates the potential impact of age-related changes in neurometabolic-flow coupling on the responsiveness of the BOLD signal.

## Materials and Methods

### Participants

Participants were 22 younger (ages 23–33, Mean age: 28.2 years; *n* = 11, six female) and older (ages 53–72, Mean age: 60.5 years; *n* = 11, six female) individuals recruited from throughout the greater Dallas–Fort Worth metropolitan area. Advertisements were posted in several locations, including the world-wide web, local community centers, and the University of Texas at Dallas (UTD) and the University of Texas Southwestern Medical Center (UTSWMC) campuses. All advertisements were approved by the UTSWMC and UTD Institutional Review Boards (IRBs).

Participants were physically and cognitively healthy. That is, they did not report any history of heart, lung, neurologic, or psychiatric difficulties and were not taking blood pressure medications, diuretics, or psychotropic/psychoactive medications. Participants were cognitively intact based on assessments during the telephone prescreening (de Jager et al., 2003) and in person on the day of the scans (Folstein et al., 1975), and they were screened and deemed safe within the 3-T MR scanning environment. Participants were compensated at the rate of $100 for their time, which was approximately $50/h, including time to consent using a form approved by both the UTSWMC and UTD IRBs.

### Visual stimuli

We considered that older participants might require differing amounts of activation compared to younger participants based upon the state of affairs prior to evoked stimulation. As noted in both Pasley et al. (2007) and Shulman et al. (2007), a condition within an experiment can be used as a baseline condition for purposes of comparison. We utilized fixation as one baseline condition and parafoveal stimulation (i.e., the use of parafoveal stimulation to artificially depress baseline activation within peripheral visual areas) as a second baseline condition. By utilizing both baselines, we were able to assess changes from two different levels of activation within parafoveal cortex.

**Figure 1:**
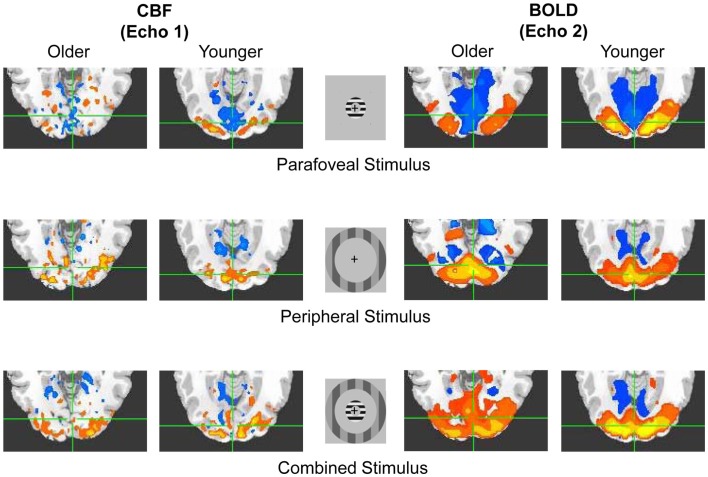
**Basic illustration of the Experiment**. Representative older and younger positive (hot color) and negative (cool color) activations in response to flashing annuli (peripheral, parafoveal, and combined stimulus types) for both CBF (i.e., echo 1) and BOLD (i.e., echo 2) signals. Signal change is shown within the overlapping anatomical and functional ROIs within visual cortex. Similar ROIs were obtained within motor cortex in response to button presses (see Materials and Methods).

Flashing sinusoidal grating annuli were presented to activate or depress activity within visual areas corresponding to the peripheral visual field (Pasley et al., 2007; Hutchison et al., 2012; cf. Figure 1). Three types of visual stimuli were presented: parafoveal stimuli (1.7–3.3° eccentricity, drift temporal frequency = 6 Hz, spatial frequency = 1 cycle/degree, 80% contrast), peripheral stimuli (6.8–9.9° eccentricity, drift temporal frequency = 6 Hz, spatial frequency = 1 cycle/degree, 25% contrast), and combined stimuli (parafoveal stimulus onset followed by peripheral stimulus onset after a 20-s delay; both images were maintained in view simultaneously after peripheral stimulus onset). Stimuli were presented in three runs, with two blocks of each stimulus type per run, and were randomized in order within each run. Thirty second fixation periods were spaced temporally between stimulus presentation blocks. Across runs, there were six instances of each type of stimulus block. Each run lasted a total of 600 s (i.e., 10 min).

A fixation cross was presented in the center of the screen throughout the visual task. It changed in luminance every 3–7 s (mean time between changes = 5 s; 118–120 luminance changes per run). Luminance changes were programed using red, green, blue (RGB) color formatting, which involves three integers representing the amounts of each of the three colors represented on the screen. By always maintaining all three components of the color equal to one another (i.e., R = G = B), the color of the fixation cross appeared as lighter and darker shades of gray. Luminance changes are henceforth discussed in terms of the change of single color values, given that all RGB values were equal for the fixation cross. These luminance changes were generally small (i.e., 20–30 U; across three runs, *n* = 183), but medium (50–90 U; *n* = 111) and large (120–240 U; *n* = 63) luminance changes were also included. This variability served to keep the task challenging enough to maintain attention at the desired fixation point, but easy enough for participants to make many knowingly accurate judgments.

### Hypercapnia materials

*R*2* (i.e., transverse relaxation rate of tissue water) is affected by blood oxygenation and is the sum of R2 (i.e., intrinsic spin-spin relaxation), *R*2′ due to non-heme field inhomogeneity (e.g., imperfect shimming, iron content), and R2′ due to deoxyhemoglobin (i.e., *M*). It is well known (see Davis et al., 1998; Hoge et al., 1999a) that only this last term is associated with BOLD signal amplitude. *M* reflects both the amount of deoxyhemoglobin related to venous cerebral blood volume (i.e., how much venous blood a voxel contains) and the oxygenation level of the venous blood. It is therefore expected that *M*, which represents the maximum BOLD signal one can get from a particular voxel, is the most relevant parameter for BOLD modulation. Because CO_2_ delivery induces vasodilation, washes out deoxyhemoglobin, and increases venous oxygenation, utilization of a hypercapnia task allowed us to infer *M* (Davis et al., 1998; Hoge et al., 1999a), which has been shown to vary across cortex (Gauthier and Hoge, 2013), and allowed for the calculation of ΔCBF/ΔCMRO_2_.

Hypercapnia was induced by delivering a 5% CO_2_ solution (balanced with 21% O_2_ and 74% N_2_; contained in a Douglas bag) via a two-way non-rebreathing valve/mouthpiece combination (Hans Rudolph, 2600 series, Shawnee, KS, USA). A capnograph device (Capnogard, Model 1265, Novametrix Medical Systems, CT, USA) was used to monitor end-tidal CO_2_, and a pulse oximeter (MEDRAD, Pittsburgh, PA, USA) was used to monitor breathing rate, heart rate, and arterial oxygenation saturation. Data values collected from both devices were recorded on a tablet PC using the HyperTerminal program (Private Edition, Version 6.3, by Hilgraeve, Monroe, MI, USA).

### Apparatus and scanning parameters

Scanning followed the same procedure as that of Hutchison et al. (2012). Imaging was conducted at the UTSWMC Advanced Imaging Research Center on a 3-T MRI system (Philips Medical Systems, Best, Netherlands). High resolution anatomical data were acquired using a T1-weighted magnetization-prepared rapid acquisition of gradient echo (MPRAGE) pulse sequence (Brant-Zawadzki et al., 1992). Calibrated fMRI using ASL allowed us to acquire both CBF (echo 1) and BOLD (echo 2) signals in a simultaneous, interleaved fashion.

A pseudo-continuous ASL sequence (Garcia et al., 2005) was used to acquire the calibrated fMRI data (visuomotor, hypercapnia) with echo times at TE1 = 11 ms and TE2 = 30 ms (Flip angle = 90°, 16 slices, 5 mm thick, orientation transverse, slice around calcarine sulcus linearly from bottom to top, TR = 4 s, 150 volumes). Global baseline CBF was measured in sagittal sinus (phase contrast voxel size 0.45 mm × 0.45 mm × 5 mm, maximum velocity 80 cm/s, duration 30 s; note that hypercapnia-induced CBF increases are comparable for sagittal sinus and feeding arteries, which perfuse the entire brain; Aslan et al., 2010) using phase contrast scans that were run immediately prior to and immediately following the hypercapnia sequence, representing normocapnic and hypercapnic conditions, respectively. Phase contrast MRI data were used to normalize ASL signals as described previously (Aslan et al., 2010). Scans were acquired in the following sequence: high resolution anatomical MPRAGE, three runs of the visuomotor task, normocapnic phase contrast, hypercapnia, hypercapnic phase contrast.

### Procedure

As in Hutchison et al. (2012), the high resolution anatomical MPRAGE was acquired first, followed by three runs of the visuomotor task. Participants were asked to ignore the flashing annuli but to quickly press and release both buttons (incorporated into hand-held button boxes) every time they noticed the fixation cross changing in luminance – that is, appearing lighter or darker. The luminance judgment task served to maintain the participants’ focus on the center of the screen so that positive response visual areas could be located functionally using calibrated fMRI responses to the flashing annuli. The button press portion of the task allowed us to assess functional changes in motor cortex.

At the conclusion of the visual stimulus runs, the patient table was withdrawn from the bore of the scanner and the mouthpiece for the hypercapnia portion of the experiment was placed into the participant’s mouth. The participant wiped the exterior of his or her nose with a moist towelette, the nose clip was placed on the participant’s nose, and it was verified that the participant could breathe comfortably and that the non-rebreathing valve was working properly. Button boxes were removed and a pulse oximeter finger monitor was clipped onto a finger of the participant’s left hand. An emergency squeeze bulb was positioned near the right hand such that the participant could easily squeeze it without having to move or look for the bulb. The patient table was returned to the scanner bore and the hypercapnia experiment procedure was executed (4 min of room air followed by 6 min of CO_2_), flanked by two, 30 s phase contrast scans (normocapnic and hypercapnic, respectively). A researcher stayed inside the magnet room throughout the phase contrast and hypercapnia portions of the experiment to manually switch the valve to control the breathing of air (either room air or CO_2_ bag). At the termination of the final scan, the bag valve was returned to room air, the patient table was withdrawn from the bore of the scanner, and the hypercapnia equipment was removed from the participant.

### Data pre-processing

Data were transformed from Philips PAR/REC format into SPM (Statistical Parametric Mapping, Wellcome Department of Imaging Neuroscience, University College London, London, UK) Analyze format using the GUI FOR R2A Rec-to-Analyze Converter (Hermans and Neggers, GUI for R2A, version 2.1 released March 2006, modified February 2008, Helmholtz Institute, Utrecht University, Department of Brain Research, University Medical Center, Utrecht, Netherlands). Files were then spatially pre-processed in SPM using the Realign-Estimate-Reslice algorithm for each echo and run. In-house MATLAB^®^ (Version 7.4, Mathworks, Natick, MA, USA) code separated echo 1 and echo 2 into separate Rec files for each echo and run, and the new Rec files were then read into AFNI (Analysis of Functional NeuroImages; Cox, 1996) where the data were registered to the individual’s MPRAGE space and aligned to the first brick of the first run of echo 2; dummy scans prevented the need to discard the first few volumes. Parameters were saved and applied to all runs for both echo 2 and echo 1. Labeled images were subtracted from control images in echo 1 to obtain CBF weighted images. Echo 2 data were subjected to a low pass temporal Fourier filter (cutoff frequency = 0.05 Hz) and were then spatially smoothed (FWHM = 8 mm). Noise was cleared from outside the head. A mask was then generated to restrict analyses to areas representing visual cortex – specifically, we utilized a morphometric approximation of Brodmann Areas (BAs; Brodmann, 1909/2006) 17, 18, and 19. BAs were defined by the demarcation of landmarks in each individual’s native space using Caret (Van Essen et al., 2001) as detailed in Hutchison et al. (2012). Functional activation in response to the visual stimuli was used to narrow region of interest (ROI) selection as described below.

### Data analyses, visual data

Echo 1 and echo 2 data, reflecting CBF and BOLD respectively, were analyzed using the 3dDeconvolve command to conduct a regression analysis in AFNI (Cox, 1996). Beta values, along with their associated *p* values, were generated for each individual for each stimulus condition for each echo.

For further analysis, ROI selection was based on functional activation overlap between CBF and BOLD but was restricted to the morphometrically defined visual cortical areas BAs 17, 18, and 19 (see above). In light of evidence suggesting that the dependent measures of interest might be less prone to bias if blood flow is taken into account and ROIs are sufficiently large (Leontiev et al., 2007), we required overlap of CBF and BOLD activation but selected a generous thresholding measure (Poline et al., 2006) such that *t* values of 1.00 were adequate for positive and negative response conditions, and the combined condition had no statistical thresholding. (Positive responses were considered to be signal increases from the baseline condition of interest; negative responses were considered to be signal decreases from the baseline condition of interest.) Thus, in order for a voxel to be retained within the ROI, it had to: (1) react positively to the peripheral stimulus for both CBF and BOLD, (2) react negatively to the parafoveal stimulus for both CBF and BOLD, (3) react positively to the combined stimulus for both CBF and BOLD, and (4) be contained within the morphometrically defined BAs 17, 18, and 19 (cf. Figure 1). ROIs for individuals ranged from 6 to 269 voxels (older: 6–136 voxels, mean = 65.36, SEM = 12.45; younger: 18–269 voxels, mean = 128.70, SEM = 24.62). Concerns regarding biases introduced by ROI size differences between the groups are obviated by our results, which indicate a pattern opposite to previous observations that smaller functional ROIs tend to be associated with larger ΔCBF/ΔCMRO_2_ ratios (Leontiev et al., 2007), and thus typically larger BOLD responses. Restated, although a negative association between ROI size and ΔCBF/ΔCMRO_2_ would be expected, we observed no such association from fixation (Spearman ρ = 0.37, *p* = 0.100) nor from parafoveal stimulation, where we observed the opposite result – that is, a positive association between ROI size and ΔCBF/ΔCMRO_2_ (Spearman ρ = 0.47, *p* = 0.030; FDR = 0.08). Age group was not a factor in these results (all *p*s > 0.05). This suggests that our results are not a simple reflection of ROI size differences. All results reported here were averaged over the ROI for the stimulus condition of interest unless specifically stated as being calculated over the whole brain.

Beta values from the regression analysis were adjusted to reflect percent signal change from fixation (for echoes 1 and 2, respectively) and were then masked by the ROI selection that included the overlap area between CBF and BOLD activations to obtain mean values per stimulus condition for older and younger groups. Additional statistical analyses were conducted using SAS^®^ software program (Version 9.1, SAS Institute, Cary, NC, USA) outside of brain space.

### Data analyses, hypercapnia data

Data in echo 1 were used to obtain CBF weighted images and data in echo 2 represented the BOLD images, as described above. The ROI masks defined in the visual fMRI runs were applied to the hypercapnia data to obtain time courses of CBF and BOLD signals. The CBF and BOLD percent signal changes were calculated comparing the average hypercapnia signal (volumes 91–150) to the average normocapnia signal (volumes 1–60).

Continuing to use in-house Matlab code, we used the Davis et al. (1998) and Hoge et al. (1999a) model to estimate *M* (a calibration constant necessary for the calculation of CMRO_2_; Davis et al., 1998) from calibrated fMRI hypercapnia data:

(1) $$M=\frac{\frac{\Delta \text{BOLD}}{\text{BOL}{\text{D}}_{0}}}{\left(1-{\left(1+\frac{\text{CBF}}{\text{CB}{\text{F}}_{\text{0}}}\right)}^{\alpha -\beta }\right)},$$

with the assumption that CMRO_2_ and neural activity are not significantly affected by hypercapnic conditions (Hoge et al., 1999b). We assumed α = 0.38 (Grubb et al., 1974) and β = 1.33 (Lu and van Zijl, 2005); Hoge et al. (1999a) demonstrated that although estimates of α and β affect asymptotic extrapolations of *M*, these values do not significantly impact results in the range of human imaging studies, and so the utilization of estimates for α and β was deemed adequate. *M* was calculated both over the ROI only and over the entire brain. There is some evidence that age-related differences in *M* can lead to differences in the BOLD response (Ances et al., 2009, but, see Hoge et al., 1999a). To be thorough, we investigated this possibility and did not find significant differences in *M* values between older and younger groups over the functionally derived ROI [older: mean *M *= 0.12, SEM = 0.01; younger: mean *M *= 0.10, SEM* *= 0.02; *t*(20) = 0.92, *p *= 0.369, *ns*]. Comparing *M* values averaged over the entire brain between the older and younger groups was similarly non-significant. Due to recent uncertainty in the literature regarding the appropriate value that should be used for α (e.g., Gauthier and Hoge, 2012), we also calculated *M* using α = 0.10. This low α estimate likewise yielded non-significant age-related *M* differences [older: mean *M* = 0.12, SEM = 0.01; younger: mean *M* = 0.15, SEM = 0.03; *t*(13.473) = −0.69, *p* = 0.502, *ns*] and allowed us to feel confident that α = 0.38 was sufficient for our visual analyses.

Finally, using *M* from the visually derived ROI, we calculated ΔCMRO_2_ and ΔCBF/ΔCMRO_2_ from fixation and from parafoveal stimulation for each individual. (One younger participant had a ΔCBF/ΔCMRO_2_ ratio more than 2.5 standard deviations greater than the remainder of the group. In order to present a clearer picture of the mean group values, this participant’s ΔCBF/ΔCMRO_2_ ratio data are not included in the analyses presented in the Section “Results,” although the inclusion of this participant’s data did not alter the significance of the results.) Similar ratios were calculated for BOLD and CBF data. The Davis et al. (1998) and Hoge et al. (1999a) model was also the basis for these calculations. Values reported in the paper use individual *M* values (cf. Chiarelli et al., 2007a) even though calculations including mean *M*s across the experiment or across groups did not alter the results.

### Data analyses, motor cortex data

Data from motor cortex were analyzed similarly to the data from visual cortex, with a few minor adjustments based on the difference of the task. In order for a voxel to be retained in the analyses, it had to: (1) react positively to button presses for both CBF and BOLD using a cutoff *t* value of 1.00, and (2) be contained within the morphometrically defined BA 4, representing primary motor cortex. Because some of the luminance changes were subtle (as described above under Visual Stimuli) and were missed by several of the participants, we analyzed only the luminance change button presses that co-occurred with a change in the flashing annuli, as these changes were typically the most easily detected. A general linear model (GLM) was sufficient to model the data, as we collapsed across luminance change and annulus type. *M* values were calculated over the area of overlap between BA 4 and the hypercapnic data acquisition and did not differ significantly between older and younger groups [older: mean *M* = 0.09, SEM = 0.03; younger: mean *M* = 0.07, SEM = 0.02; *t*(19) = 0.35, *p* = 0.733, *ns*]; using α = 0.10 did not affect this result [older: mean *M* = 0.07, SEM = 0.02; younger: mean *M* = 0.06, SEM = 0.02; *t*(19) = 0.35, *p* = 0.733, *ns*]. One older participant did not retain any significant voxels within BA 4. An additional older participant’s functional values were determined to be outliers in the opposite direction (i.e., greatly exaggerated responses); however, the removal of the outlying data did not markedly affect the pattern of results. Data for the remaining 20 participants are therefore represented for the motor cortex analyses throughout the remainder of the paper. There was no significant association between ROI size and ΔCBF/ΔCMRO_2_ (Spearman ρ = −0.21, *p* = 0.361).

### Neurometabolic-flow coupling and neural efficiency simulation

We speculated that reduced vascular responsiveness to neural metabolic demand would lead to variation in ΔCBF/ΔCMRO_2_ with variation in task demand. We examined the effect of vascular responsiveness, indexed by age-differential ΔCBF/ΔCMRO_2_ coupling ratios, on BOLD changes for different levels of ΔCMRO_2_. BOLD was plotted relative to ΔCMRO_2_ for lower and higher neurometabolic-flow coupling ratios (approximating those for old and young, respectively). We could thereby visualize the effect of age-related changes in ΔCBF/ΔCMRO_2_ on the BOLD signal. Previous research has shown that, for a wide range of ΔCMRO_2_ and ΔCBF, these two metrics are tightly coupled in a linear fashion in younger adults (e.g., Stefanovic et al., 2004). This coupling appears to be adversely affected in aging, yielding age-related reductions in ΔCBF/ΔCMRO_2_ for older compared to younger adults (Hutchison et al., 2012). Using the theoretical relationship between ΔCMRO_2_, ΔCBF, and ΔBOLD, we investigated whether such changes in ΔCBF/ΔCMRO_2_ would differentially affect the rate of changes in the BOLD signal given (Davis et al., 1998; Hoge et al., 1999a):

(2) $$\frac{\Delta \text{BOLD}}{\text{BOL}{\text{D}}_{0}}=M\left(1-{\left(\frac{\text{CMR}{\text{O}}_{\text{2}}}{\text{CMR}{\text{O}}_{\text{2}}{|}_{0}}\right)}^{\beta }{\left(\frac{\text{CBF}}{\text{CB}{\text{F}}_{0}}\right)}^{\alpha -\beta }\right)$$

Changes in the BOLD signal were considered across different levels of ΔCMRO_2_ relative to baseline conditions (fixation and parafoveal stimulation).

## Results

*A priori* hypotheses of age-related changes in ΔCBF/ΔCMRO_2_ ratios and their relationships to behavior were tested using planned comparisons (cf. Keppel and Wickens, 2004). For additional tests we calculated, using False Discovery Rate (FDR) theory (Benjamini and Hochberg, 2000), that we would expect 0.85, or roughly between 0 and 1, of these tests to falsely reject the null hypothesis (0.05 × 17 = 0.85). For these tests, we rejected the null hypothesis at *p* < 0.05, FDR < 0.05. Thus it is probable that our tests correctly rejected our null hypotheses, particularly given that the results portray a consistent picture. For the sake of clarity, the FDR is reported in addition to *p* when *p* < 0.05 and FDR < 0.10.

Blood-oxygen-level-dependent data and *M* values were normally distributed. All other variables deviated from normality (based on the Shapiro–Wilk *W* statistic, *p*s < 0.05). For example, ΔCBF/ΔCMRO_2_ was not normally distributed when assessed from visual fixation (*W* = 0.74, *p* < 0.0001), parafoveal stimulation (*W* = 0.81, *p* = 0.0008), or within motor cortex (*W* = 0.72, *p* < 0.0001). Under such conditions of non-normality we used non-parametric (i.e., Kruskal–Wallis, Signed rank, or Spearman ρ) tests to assess group differences and associations between variables.

### Behavioral results

Older participants were significantly slower than younger participants in responding to fixation cross luminance changes [older: mean RT = 687.66 ms, SEM* *= 34.88; younger: mean RT = 553.93 ms, SEM* *= 31.21; Kruskal–Wallis (K–W) χ^2^(1) = 6.06, *p* = 0.014]. Older participants were also less accurate than their younger counterparts [older: mean proportion correct = 0.69, SEM* *= 0.05; younger: mean proportion correct = 0.83, SEM* *= 0.02; K–W χ^2^(1) = 4.55, *p* = 0.033; FDR = 0.08]. RT was negatively associated with proportion correct (Spearman rho (ρ) = −0.67, *p *= 0.0007), yielding no support for a speed-accuracy trade-off.

### Visual experiment

#### Blood-oxygen-level-dependent

We first tested the *a priori* hypothesis of an age-related difference in the BOLD response within visual cortex, as the etiology of this difference was the topic under study. A mixed model incorporating group (older and younger), stimulus conditions of interest (from fixation: parafoveal, peripheral, and combined; from parafoveal stimulation: combined), and a group by stimulus condition interaction, was used to assess differences in the BOLD response in terms of proportion signal change. In addition to differences solely based on stimulus type [*F*(3, 60) = 288.47, *p* < 0.0001], there was a significant interaction between age group and stimulus type [*F*(3, 60) = 5.21, *p* = 0.003]. Most pronounced was the difference between older and younger groups when responding to the combined stimulus from parafoveal stimulation [older: least Squared Mean (LSM) = 0.0087, SEM = 0.0006; younger: LSM = 0.0114, SEM = 0.0006; difference estimate (*de*) = −0.0027; *t*(48.8) = −3.00, *p* = 0.004], but this group difference in responding to the combined stimulus was also significant from fixation [older: LSM = 0.0062, SEM = 0.0006; younger: LSM = 0.0081, SEM = 0.0006; *de* = −0.0019; *t*(48.8) = −2.08, *p* = 0.043; see Table 1]. Collapsing across all stimulus types, the younger group had a marginally greater BOLD response than the older group [*de* = −0.0013, *t*(20) = −1.85, *p* = 0.079].

**Table 1:** **Mean proportion signal change (BOLD, ΔCBF/ΔCMRO_2_, ΔCBF, ΔCMRO_2_) within visual cortex and motor cortex, from baseline (fixation and non-motor response periods, respectively)**.

Cortical area, task	BOLD*		ΔCBF/ΔCMRO_2_*		ΔCBF		ΔCMRO_2_*	
	Older	Younger	Older	Younger	Older	Younger	Older	Younger
Visual cortex, response to flashing annuli (SEM)	0.0059 (0.0008)	0.0073 (0.0007)	1.8844 (0.0639)	2.3977 (0.2360)	0.3621 (0.0542)	0.3267 (0.0302)	0.1969 (0.0336)	0.1327 (0.0194)

	**BOLD**		**ΔCBF/ΔCMRO_2_**		ΔCBF*		ΔCMRO_2_*	
	**Older**	**Younger**	**Older**	**Younger**	**Older**	**Younger**	**Older**	**Younger**

Motor cortex, response to flashing annuli (SEM)	0.0016 (0.0001)	0.0022 (0.0003)	1.6275 (0.0166)	1.6639 (0.0276)	0.8788 (0.1231)	0.5624 (0.0721)	0.5354 (0.0691)	0.3361 (0.0393)

*Difference between groups, *p* < 0.05.

#### ΔCBF/ΔCMRO_2_ ratio

To test the *a priori* hypothesis that ΔCBF/ΔCMRO_2_ differences could mediate age-related changes in the BOLD response, ΔCBF/ΔCMRO_2_ was calculated for each individual from both resting and parafoveal stimulation conditions. ΔCBF/ΔCMRO_2_ was significantly greater for the younger group than the older group, both from fixation [older: mean = 1.88, SEM = 0.06; younger: mean = 2.40, SEM = 0.24; K–W χ^2^(1) = 4.17, *p *= 0.041; see Table 1], and from parafoveal stimulation [older: mean = 1.69, SEM = 0.05; younger: mean = 2.00, SEM = 0.12; K–W χ^2^(1) = 4.46, *p *= 0.035]. These results support the hypothesis that age-related BOLD signal changes are the consequence of differences in the ratio of cerebral perfusion to oxygen metabolic rate (i.e., ΔCBF/ΔCMRO_2_) between the older and younger groups, and these age-related differences occur regardless of baseline stimulation condition (i.e., fixation or parafoveal).

#### Components of the ΔCBF/ΔCMRO_2_ ratio

Significant differences in the CBF response would indicate that age-related differences in the BOLD response are at least partially due to age-related changes in cerebral perfusion (i.e., blood flow). As with the BOLD data, we utilized a mixed model incorporating group, stimulus conditions of interest (from fixation: negative, positive, and combined; from parafoveal stimulation: combined), and a group by stimulus condition interaction to assess differences in proportion signal change (see Gupta et al., 2006, regarding the robustness of a balanced, mixed model to non-normality). As seen in Table 1, the ΔCBF difference between older and younger groups was not significant [*F*(1, 20) = 0.20, *p *= 0.659], indicating that age-related differences in ΔCBF/ΔCMRO_2_ were not caused by differences in the ΔCBF response. Additionally, there were minimal differences in “absolute” (i.e., change from fixation) ΔCBF in the older compared to the younger group, both from fixation [K–W χ^2^(1) = 0.24, *p* = 0.622] and parafoveal stimulation [K–W χ^2^(1) = 0.05, *p *= 0.818].

We also sought to determine if there were age-related differences in the ΔCMRO_2_ component of ΔCBF/ΔCMRO_2_. We observed age-related ΔCMRO_2_ differences in “absolute” measures (i.e., change from fixation) when measured from parafoveal stimulation [K–W χ^2^(1) = 4.28, *p* = 0.039; FDR = 0.08]. Additionally, we observed greater “incremental” (i.e., change from respective baseline stimulation condition – that is, either fixation or parafoveal stimulation) evoked ΔCMRO_2_ from parafoveal stimulation than from fixation [from parafoveal stimulation: mean = 0.30, SEM = 0.02; from fixation: mean = 0.16, SEM = 0.02; Signed rank *S* = 126.50, *p* < 0.0001]; this effect was also found for BOLD and ΔCBF (*p*s < 0.0001). However, only the BOLD response showed smaller incremental evoked responses for the older group compared to the younger group [older: LSM = 0.0073, SEM* *= 0.0007; younger: LSM = 0.0094, SEM = 0.0007; assessed using a mixed model, *de* = −0.0021, *t*(20) = −2.09, *p *= 0.050; FDR = 0.09], indicating that age-related differences in ΔCMRO_2_ are not attributable to baseline stimulation condition differences. In sum, comparing differences between older and younger participants on the constituents of the BOLD response within visual cortex revealed minimal differences in ΔCBF but significant age-related differences in ΔCMRO_2_ (see Table 1). This change in ΔCMRO_2_ affected significant change in ΔCBF/ΔCMRO_2_.

#### ΔCBF/ΔCMRO_2_: relationships to behavior

**Figure 2:**
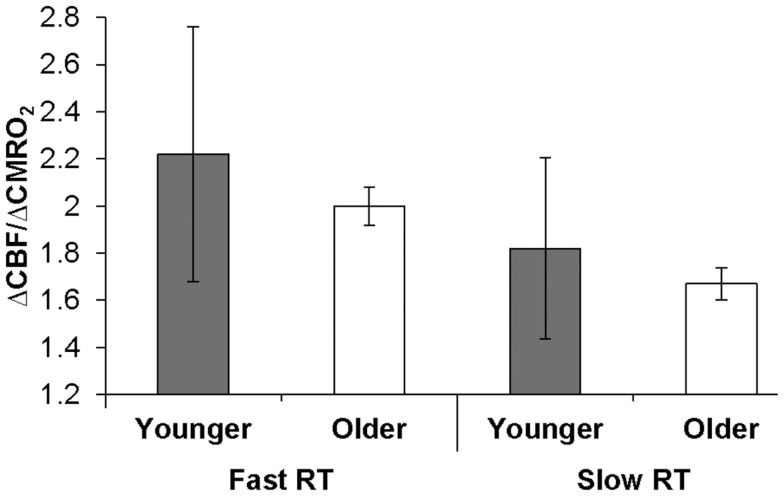
**Relationship of ΔCBF/ΔCMRO_2_ to behavior within visual cortex**. Older and younger participants were designated as fast or slow responders based on median reaction time (RT) within each group. Error bars represent the semi-interquartile range. The resulting median ΔCBF/ΔCMRO_2_ ratios for each group from fixation illustrate the significant association between ΔCBF/ΔCMRO_2_ and RT, which was calculated across groups.

The behavioral significance of age-related ΔCBF/ΔCMRO_2_ differences could be demonstrated if we observed relationships between this physiologic factor and individual subject performance. A Spearman ρ test of association across older and younger groups confirmed our *a priori* hypothesis that RT was significantly negatively associated with ΔCBF/ΔCMRO_2_ from fixation (ρ = −0.49, *p *= 0.025; and from parafoveal stimulation, ρ = −0.43, *p *= 0.053). Figure 2 illustrates this relationship when broken out by group (older or younger) and RT (slower or faster, as determined by within-group median split). Likewise, a Spearman ρ test of association confirmed our *a priori* hypothesis that proportion correct was positively associated with ΔCBF/ΔCMRO_2_ from fixation (ρ = 0.46, *p *< 0.038). Neither RT nor proportion correct was associated with the ΔCBF or ΔCMRO_2_ components separately. These results support the hypothesis that age-related behavioral indices – i.e., slowing of RTs and reduced proportion correct – are significantly associated with age-related reductions in ΔCBF/ΔCMRO_2_.

### Motor cortex analyses

Because ΔCBF and ΔCMRO_2_ can vary independently across cortex, we additionally analyzed BOLD, ΔCBF/ΔCMRO_2_, ΔCBF, and ΔCMRO_2_ data from the button press task within motor cortex (see Table 1). A GLM indicated that older participants had only a marginally smaller BOLD response within motor cortex than did younger participants [older: mean = 0.0016, SEM = 0.0001; younger: mean = 0.0022, SEM = 0.0003; *F*(1, 18) = 3.33, *p* = 0.085, *ns*] in response to button press stimulation. Also in contrast to the visual cortex results, ΔCBF/ΔCMRO_2_ was statistically similar in older and younger groups [older: mean = 1.63, SEM = 0.02; younger: mean = 1.66, SEM = 0.03; K–W χ^2^(1) = 1.39, *p* = 0.239, *ns*]. Breaking the ratio down into its constituent components yielded increases in ΔCBF for the older group compared to the younger group [older: mean = 0.8788, SEM = 0.1231; younger: mean = 0.5624, SEM = 0.0721; K–W χ^2^(1) = 6.10, *p* = 0.014]. There were also increases in ΔCMRO_2_ for the older group compared to the younger group [older: mean = 0.5354, SEM = 0.0691; younger: mean = 0.3361, SEM = 0.0393; K–W χ^2^(1) = 6.88, *p* = 0.009].

In sum, comparing differences between older and younger participants on the constituents of the BOLD response within motor cortex revealed age-related differences in both ΔCBF and ΔCMRO_2_. Changes in both components of the ratio offset one another, leading to an absence of significant differences in ΔCBF/ΔCMRO_2_. This difference was reflected in age-equivalent BOLD responding.

### Neurometabolic-flow coupling and neural efficiency simulation

**Figure 3:**
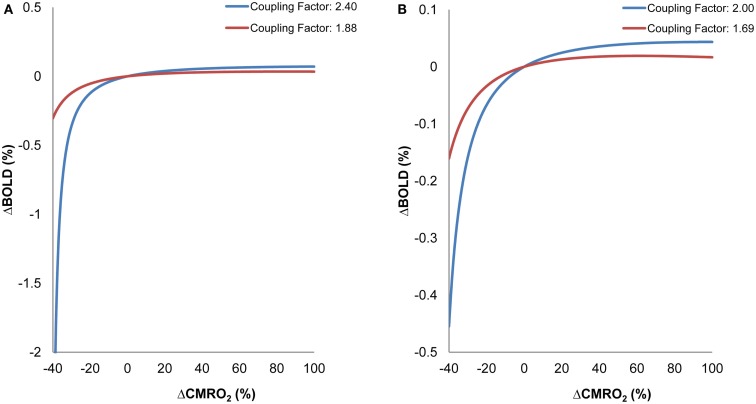
**Simulation Results: BOLD as a function of ΔCMRO_2_ for different ΔCBF/ΔCMRO_2_ coupling ratios within visual cortex**. **(A)** ΔCBF/ΔCMRO_2_ coupling ratios derived from fixation for older (red) and younger (blue) participants. **(B)** ΔCBF/ΔCMRO_2_ coupling ratios derived from parafoveal stimulation for older (red) and younger (blue) participants. Variations in BOLD and CMRO_2_ were calculated relative to a baseline representing an intermediate activity level for each condition (fixation, parafoveal stimulation).

Figure 3 shows BOLD signal variability as a function of ΔCMRO_2_. The simulation analysis revealed that the BOLD signal, in general, is less responsive to ΔCMRO_2_ changes in the older group than in the young group for both fixation and parafoveal stimulation conditions (Figures 3A,B). It can be seen that, for reduced levels of CMRO_2_ (i.e., conditions demanding lower neuronal activity relative to a baseline condition), the older group showed less BOLD signal change than the younger group. However, for increased levels of CMRO_2_ (i.e., conditions demanding higher neuronal activity relative to a baseline condition), the younger group showed greater BOLD signal change than the older group. In sum, decreased coupling ratios in the older group led to less responsiveness in BOLD signal in either direction (i.e., increased and decreased CMRO_2_) compared to the younger group. These interaction patterns suggest that, for identical changes in ΔCMRO_2_ (which more directly reflect the level of neuronal activity), differing patterns of BOLD signal change are expected in older and younger groups, due to variations in the ΔCBF/ΔCMRO_2_ coupling ratios. In relation to fMRI findings in the aging literature, it is worth noting that these BOLD changes are not exclusively a result of changes in levels of underlying neuronal activity. Instead, the BOLD changes are significantly affected by age-related changes in the relationship between ΔCBF and ΔCMRO_2_.

## Discussion

Using a visuomotor task, we observed age-related changes in neurometabolic-flow response patterns in both visual and motor cortices using calibrated fMRI. Specifically, within visual cortex, we found equivalent ΔCBF in the presence of age-related increases in ΔCMRO_2_. This reduction in the ΔCBF/ΔCMRO_2_ ratio resulted in paradoxical age-related decreases in BOLD for older adults relative to younger adults, even in the face of increases in ΔCMRO_2_, a more direct index of neural activity (cf. Hyder, 2004). Additionally, these age-related ΔCBF/ΔCMRO_2_ ratio decreases were related to RT, suggesting that age-related slowing observed in visual processing tasks might result from less efficient neural cell assemblies. Within motor cortex, we found age-related increases in both ΔCBF and ΔCMRO_2_. Changes in both aspects of the ΔCBF/ΔCMRO_2_ ratio offset one another, yielding age-equivalence in ΔCBF/ΔCMRO_2_ and in BOLD signal within motor cortex. A simulation illustrated how age-related changes in BOLD might reflect differential changes in ΔCBF/ΔCMRO_2_ instead of simply reflecting changes in neural activity as indexed by ΔCMRO_2_. These results suggest that age-comparative studies relying solely on BOLD signal might be systematically misinterpreted depending on the cortical area, cognitive task, and the age group under investigation.

Age-differences in the BOLD response arise from the variable relationship between ΔCBF and ΔCMRO_2_; ΔCBF increases lead to BOLD amplitude increases, whereas ΔCMRO_2_ increases lead to BOLD amplitude decreases (Buxton et al., 2004). This complex relationship could explain much of the variability between studies comparing cerebrovascular characteristics of older and younger adults (Ross et al., 1997; Taoka et al., 1998; D’Esposito et al., 1999; Huettel et al., 2001; Restom et al., 2007; Ances et al., 2009; Lu et al., 2011). The present results, showing age-related ΔCBF/ΔCMRO_2_ alterations in primary visual regions but no age-related ΔCBF/ΔCMRO_2_ alterations in primary motor regions, suggest that between-study variance in previous results might be due to age-related regional differences in ΔCBF and ΔCMRO_2_ (Heo et al., 2010; Chen et al., 2011; Lu et al., 2011). The resulting ΔCBF/ΔCMRO_2_ would lead to apparent age-equivalence – or age-differences – in the BOLD response.

There has been considerable investigation and debate regarding the ideal values for α and β parameters used to calculate *M* and ΔCMRO_2_, as an artificially large α value could significantly underestimate ΔCMRO_2_ (although biases of ΔCBF/ΔCMRO_2_ are somewhat limited if the same α value is used to estimate both *M* and ΔCMRO_2_; Chen and Pike, 2009). In particular, several studies have found α values to be smaller than Grubb et al.’s (1974) value of 0.38 that is used widely throughout the literature (e.g., Chen and Pike, 2009, 2010). Recently, Gauthier and Hoge (2012) investigated ranges of α (0.1–0.38) and β (1–2) values in terms of their effect on *M* and oxygen extraction fraction (OEF) estimates. Using three different gas solutions, Gauthier and Hoge (2012) generated maps of *M*, OEF, CBF, and CMRO_2_ across the brain and found convergent α and β values to result in only slightly different (i.e., statistically similar) *M* and oxygen extraction estimates compared to those generated using nominal α and β values. Likewise, using a paradigm similar to that of the current experiment, Pasley et al. (2007) conducted an error analysis using α = −1.0 to 0.4; concluding that their results were not dependent on precise parameter values, they elected to utilize α = 0.38 for their calculations. These two strong examples from the literature are consistent with our own observations and suggest that our results are robust.

Considering Gauthier and Hoge’s (2012) maps of *M*, OEFs, and CMRO_2_ across the brain, it is possible that α and β values vary across brain regions and/or tasks (but, see Chen and Pike, 2009, 2010, regarding statistical equivalence between brain regions). In a hypercapnia/hypocapnia experiment, Chen and Pike (2010) found α to be equivalent to 0.18 (±0.02); however, when implementing a high intensity, high-contrast visual stimulation condition much like that of the parafoveal stimulation condition in the current experiment, Chen and Pike (2009) found α = 0.31 (±0.10), which subsumes the α value of 0.38 used in the current experiment. Further, Kida et al. (2007) found α to increase along with duration of forepaw stimulation. Because of our block design and thus long-duration stimulation conditions, larger as opposed to smaller α values would be warranted in the present study. But Kida et al. (2007) also found different values of α over different CBF phases within the stimulation timeframe, complicating the matter. Unfortunately, the determination of the correct value of α is not a settled science. Continued research on this topic is certainly needed to elucidate the influence of α and β estimates upon mixed results observed in the literature. In summary, given the range of estimates available in the literature, our choice of calculation parameters was informed in two ways. First, it was informed by those conditions in the above-cited studies most analogous to our own (i.e., long-duration, high-contrast stimuli). Second, it was informed by those studies upon which our methods were most directly based, and to which we hoped to most directly compare our results (i.e., Pasley et al., 2007; Restom et al., 2007; Ances et al., 2009; Mohtasib et al., 2012).

Cross-cortex variability (Rypma and D’Esposito, 2001; Rypma et al., 2006; Rypma and Prabhakaran, 2009) has important implications for neurocognitive aging hypotheses based on BOLD signal because it suggests that regional BOLD age-differences, or equivalences, cannot be unambiguously interpreted in terms of neural activity. Further, we found age-related performance reductions (greater RT and reduced proportion correct) to be significantly associated with reduced ΔCBF/ΔCMRO_2_ within visual cortex, suggesting that alterations to ΔCBF/ΔCMRO_2_ affected the processes by which the visual stimuli were perceived.

The present results have both practical and theoretical implications. From a practical standpoint, our results implicate a specific mechanism by which age-related changes in the BOLD response to neural activity arise. That is, age-related alterations in ΔCMRO_2_, reflecting alterations in neural activity, are not accompanied by an adequate CVR (cf. Ances et al., 2009; Lu et al., 2011; Gauthier et al., 2012). Both ΔCMRO_2_ and ΔCBF, and their ratio to one another, can be affected differentially across cortex and tasks. Thus BOLD responses might be indexing different underlying physiological changes in young and old across the cortex. Specifically, decreased ΔCBF/ΔCMRO_2_ coupling ratios in the older population result in less responsiveness in BOLD signal in either direction (i.e., increased and decreased CMRO_2_) compared to the younger population. This response leads to greater BOLD signal for the older group compared to the younger group in relatively lower CMRO_2_ ranges but lower BOLD signal for old compared to young in relatively higher CMRO_2_ ranges.

Neurocognitive aging research seeks to understand the neural basis of age-related performance changes using BOLD signal as a proxy for neural activity. The BOLD response, although relatively easy to acquire, and heavily reported in the aging literature, is not so easy to interpret. Our results showed age-related ΔCMRO_2_ increases in two different brain regions. In one region (primary visual cortex), BOLD signal was reduced in the older compared to the younger group. In the other region (motor cortex), BOLD signal was equivalent between the two groups. Together these results suggest that regional BOLD age-differences, or age-equivalence, cannot be unambiguously interpreted in terms of neural activity. Thus, our results suggest that techniques such as the dual-echo ASL method applied here, that allow for the calculation of ΔCMRO_2_, are necessary, as ΔCMRO_2_ is more readily interpretable as an index of neural activity compared to the BOLD response which appears to reflect a complex of neural, vascular, and probably glial factors.

From a theoretical standpoint, the associations we observed between age-related performance reductions and reductions in the ΔCBF/ΔCMRO_2_ ratio support the hypothesis that increased oxygen demand in the aging neural apparatus, not accommodated by vascular activity, is the basis for reduced functional efficiency at the neural level and reduced processing efficiency at the cognitive level. Our hypothesis receives support from observations of age-related variability increases in ΔCBF/ΔCMRO_2_ (cf. Figure 2). Reduced BOLD signal variability in old compared to young has been documented in recent literature (e.g., Garrett et al., 2010, 2011). Consistent with our observations, Garrett et al. have suggested that this pattern might reflect inefficient processing due to reductions in the integrity of the aging neural system (Garrett et al., 2011; cf. MacDonald et al., 2009) or inflexibility in that system (Hong and Rebec, 2012).

Reductions in vascular responsiveness would have consequences for the oxygen available to neurons. Reduced oxygen availability has been associated with increased ΔCMRO_2_ such as that observed in the older adults in the present study. Infra-human positron-emission tomography (PET) studies show increased neural metabolism in limited oxygen conditions (e.g., Harik et al., 1995; Richards et al., 2007). Similar oxygen-metabolism relationships have been observed in humans (e.g., Rockswold et al., 2010; Smith et al., 2011; Xu et al., 2012). In one study, Xu et al. (2012) scanned young adults while they breathed room air (21% fraction of inspired O_2_; FiO_2_) or one of three gas mixtures that varied in O_2_ content (14% O_2_, hypoxic condition; 50 or 98% O_2_, hyperoxic conditions). The results indicated that decreases in O_2_ availability led to increases in ΔCMRO_2_, possibly owing to increased glycolytic and cytochrome oxidative activity in O_2_-depleted cells (e.g., Hamberger and Hyden, 1963). Thus Xu et al.’s (2012) results support the hypothesis that increased ΔCMRO_2_ in older neurons results from the decreased O_2_ availability afforded by reduced vascular responsiveness. The present experiment suggests that this mechanism forms the neural basis for the reduced processing efficiency that leads to slower and less accurate performance in older adults compared to younger adults, affecting among other things, their speed of response to changing hues in a central fixation cross. More research is certainly required to test the hypothesis that reduced oxygen availability to neurons in primary visual cortex leads to the broad-spread age-related declines that are observed in perceptual and visual-search tasks.

In summary, dual-echo ASL technology permitted acquisition of both ΔCBF and BOLD data in response to visuomotor stimulation. We observed that ΔCBF/ΔCMRO_2_ and BOLD varied across visual and motor cortices, and that BOLD did not accurately index neural activity in either region. A simulation illustrated how ΔCBF/ΔCMRO_2_ can alter relationships between neural activity and BOLD signal. These results suggest (1) that the calculation of ΔCMRO_2_ is necessary for accurate young-old comparisons of neural activity and (2) that the neural basis of processing efficiency declines observed in older adults’ visual task performance might be related to reductions in the oxygen available to neurons via aging vasculature.

## Conflict of Interest Statement

The authors declare that the research was conducted in the absence of any commercial or financial relationships that could be construed as a potential conflict of interest.
